# Hormonal Signaling and Follicular Regulation in Normal and Miniature Pigs During Corpus Luteum Regression

**DOI:** 10.3390/ijms26157147

**Published:** 2025-07-24

**Authors:** Sang-Hwan Kim

**Affiliations:** 1 School of Animal Life Convergence Science, Hankyong National University, 327, Jungang-ro, Ansung 17579, Gyeonggi-do, Republic of Korea; immunoking@hknu.ac.kr; 2 Institute of Applied Humanimal Science, Hankyong National University, 327, Jungang-ro, Ansung 17579, Gyeonggi-do, Republic of Korea

**Keywords:** reproduction, hormonal regulation, minipigs, porcine, follicular development

## Abstract

Reproductive efficiency in pigs is regulated by hormonal pathways that control follicular development at Day 15 of the estrous cycle, during corpus luteum regression. Miniature pigs are extensively employed as human-relevant models in biomedical research, yet their reproductive characteristics during mid-luteal regression remain inadequately characterized, limiting assessments of their translational reliability. Differences in follicular morphology, hormonal signaling, and vascular development may underlie their lower fertility compared to conventional pigs. In this study, follicular development after corpus luteum formation was compared between conventional pigs and minipigs using histological staining, immunofluorescence, hormonal assays, and transcriptomic profiling. The expression of VEGF, mTOR, LH, FSH, PAPP-A, and apoptosis markers was evaluated across the granulosa and thecal regions. Differential gene expression was analyzed using microarray data followed by GO categorization. Minipigs exhibited smaller follicles, reduced vascularization, and lower VEGF and MMP activity compared to conventional pigs. Expression of LH and PAPP-A was higher in conventional pigs, while minipigs showed relatively elevated E2 and FSH levels. Transcriptomic data revealed greater upregulation of cell-survival- and angiogenesis-related genes in conventional pigs, including genes involved in IGF pathways. Apoptosis and poor extracellular matrix remodeling were more pronounced in minipigs. Minipigs demonstrated impaired follicular remodeling and weaker hormonal signaling after corpus luteum formation, which likely contributed to their reduced reproductive efficiency. Understanding these species differences can guide breeding strategies and fertility management in biomedical and agricultural settings.

## 1. Introduction

The estrous cycle in pigs spans approximately 21 days, with ovulation initiated by a rapid surge in luteinizing hormone (LH) levels triggered by a negative feedback mechanism in response to elevated estrogen concentrations [1,2,3]. Ovulation typically occurs two days after the LH surge. Following ovulation, the ovaries undergo notable morphological changes, including the formation of the corpus luteum and the regression of previously developed but non-ovulated follicles [2,4].

During follicular development, the insulin-like growth factor (IGF) signaling pathway plays a central role; it is stimulated by estradiol to enhance granulosa cell metabolism and promote neovascularization [5,6]. Initially, preantral follicles lack vascular supply, but as development progresses, a vascular sheath forms within the theca layer. This leads to the establishment of two distinct capillary networks in the theca interna and externa, which are essential for nutrient transport and follicular maturation [7,8].

On day 15 of the estrous cycle, during corpus luteum regression, LH and progesterone signaling induce follicular regression, involving changes in survival factors and neovascularization [6,9,10]. At this stage, granulosa cells begin to experience functional decline and undergo apoptosis, a key mechanism in follicular atresia [9,10]. Apoptosis proceeds more rapidly in atretic follicles than in external follicular or thecal cells, and it is mediated by caspase-3 (Casp-3) activation following apoptotic ligand stimulation, which leads to cytosolic degradation and DNA fragmentation [11]. Proper regulation of this apoptotic cascade is critical for maintaining follicular homeostasis and ensuring appropriate oocyte selection for the subsequent reproductive cycle [12].

Miniature pigs are increasingly utilized as human-relevant models in biomedical research due to the physiological similarity between the two animals, yet follicular regulation immediately after corpus luteum formation remains insufficiently characterized, limiting confidence in these pigs’ translational applicability. However, minipigs exhibit distinct reproductive characteristics relative to conventional pigs, particularly regarding hormonal signaling and follicular development. Prior work [6] and more recent evidence [13] indicate that minipigs exhibit reduced reproductive efficiency, likely driven by alterations in key hormonal signaling pathways such as VEGF-mediated angiogenesis and LH receptor activity. Notably, LH receptor activity is lower in minipigs, resulting in delayed follicular maturation and compromised corpus luteum function [13]. This dysfunction is accompanied by more apoptosis and diminished follicular regeneration, further impairing reproductive capacity [14,15]. While the IGF/AKT pathway continues to support follicular growth in conventional pigs after corpus luteum formation, it remains unclear whether this survival mechanism persists to the same extent in minipigs [16].

A further distinction lies in vascular development and extracellular matrix (ECM) remodeling. Vascularization is vital for follicular growth and luteal maintenance, as it supports both nutrient delivery and hormonal signaling. In conventional pigs, VEGF-mediated angiogenesis and matrix metalloproteinase (MMP) activity facilitate rapid follicular expansion and ECM remodeling. In contrast, minipigs exhibit lower VEGF expression and MMP activity, leading to impaired vascularization and delayed follicular maturation following corpus luteum formation [17,18]. This reduced vascular support may compromise the hormonal microenvironment necessary for oocyte development [15]. Despite these observations, the molecular mechanisms underlying ECM remodeling and vascular development in minipigs remain poorly defined.

Apoptosis is a critical process in follicular atresia, yet its regulatory dynamics appear to differ between species. In conventional pigs, apoptosis is primarily confined to atretic follicles, while granulosa and thecal cells in developing follicles are sustained via IGF/AKT and VEGF signaling [14,15]. Conversely, in minipigs, apoptosis is prematurely activated after corpus luteum formation, resulting in early loss of granulosa cells and impaired follicular regeneration [13,17]. This suggests a deficiency in survival-promoting factors that may further destabilize the balance between follicular growth and regression. The role of Casp-3-mediated apoptosis in minipigs, particularly in the context of hormonal receptor interactions, remains insufficiently characterized. Clarifying these molecular pathways is crucial for understanding the mechanisms underlying delayed follicular development and suboptimal fertility in minipigs.

Therefore, this study was conducted to elucidate species-specific differences in follicular development and survival mechanisms between minipigs and conventional pigs. We analyzed LH receptor activity and IGF/AKT signaling, assessed VEGF and MMP expression related to vascularization and ECM remodeling, and evaluated the involvement of Casp-3-mediated apoptosis in regulating follicular survival.

## 2. Results

### 2.1. Differences in Follicular Development Between Minipigs and Conventional Pigs

**Figure 1:**
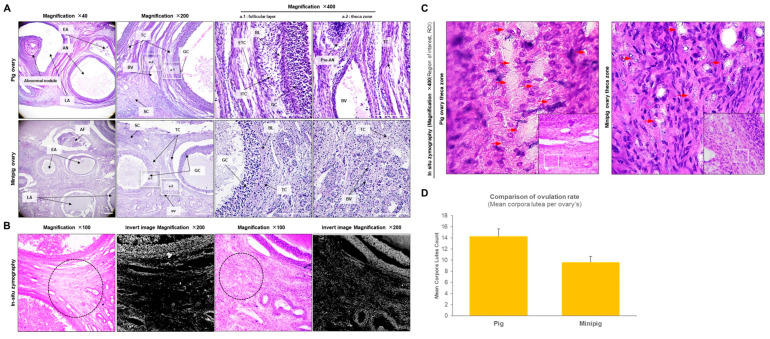
Analysis of morphological changes and differences in the cytoplasm regarding pig and minipig follicles. (**A**): These representative histochemical images compare the morphologies of large follicles in the ovaries of conventional pigs and minipigs. (**B**): The areas of granulosa, intrathecal, and extrathecal zones were quantified using in situ zymography. (**C**): To observe the theca cell region in detail in the results obtained via in situ zymography, the ROI function was used at 200× and 400× magnification. (**D**): Bars represent mean number of corpora lutea per ovary (mean ± SEM; *p* < 0.05 vs. minipigs, Student’s *t*-test). Abbreviations: AF, atretic follicle; AN, antral follicle; BL, basal layer; BV, blood vesicle; EA, early antral; ETC, extrathecal cell; GC, granulosa cell; ITC, intrathecal cell; LA, large antral; Pre-AN, preantral follicle; ROI, region of interest; SC, stroma cell; TC, theca cell. Black circles indicate areas where the reaction is prominent. Scale bar = 100 µm; magnifications = 40×, 200×, and 400×.

Figure 1A illustrates the morphological differences in follicular development between conventional pigs and minipigs. In the conventional pigs, the granulosa cell layer and basal layer were more developed, showing greater thickness and organization. Additionally, there was a higher density of intra-thecal cells and more extensive capillary dilation. In contrast, ovarian maturation appeared delayed in the minipigs, with the minipigs’ follicles maintaining preantral and early antral morphology, whereas the conventional pigs exhibited fully developed large antral follicles. These morphological disparities suggest that follicular distribution and vascular expansion are more pronounced in the ovaries of conventional pigs, resulting in significant structural differences in comparison to minipigs. In situ zymography revealed higher matrix metalloproteinase (MMP) activity in the theca cell zone of the conventional pigs in comparison to that in the minipigs (Figure 1B,C). In the conventional pigs, MMP activity was predominantly localized in the cytoplasm of the extra-thecal zone. In the minipigs, while numerous micro-vessels were observed in the extra-thecal zone, MMP activity was primarily concentrated around these micro-vessels (Figure 1C). This indicates regional differences in tissue remodeling and enzymatic activity between the two pig types. The mean ovulation rate, expressed as the number of corpora lutea per ovary, was significantly higher in the conventional pigs (14.2 ± 1.3) in comparison to the minipigs (9.6 ± 1.1; *p* < 0.05; Figure 1D). This difference corroborates the observed disparities in follicular development and reproductive efficiency between the two groups.

### 2.2. Gene Differences That May Affect Ovarian Development and Follicle Maturation in Minipigs and Normal Pigs

**Figure 2:**
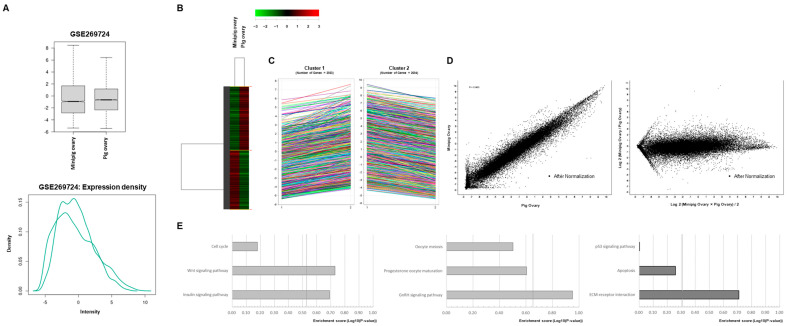
RNA microarray analysis. (**A**): Results of the unsupervised clustering analysis of minipig and control pig ovaries. (**B**): A volcano plot of all probes. The broken blue line and continuous red line denote downregulated and upregulated genes in minipig and control pig ovaries, respectively (fold-change > 3, *p* < 0.01). (**C**,**D**): Downregulated and upregulated genes in the pathway analyses of minipig and control pig ovaries, respectively. (**E**): The main mechanisms that showed the most significant differences were analyzed. The dotted line indicates the significance level (*p* < 0.05).

To compare gene expression profiles between conventional pigs and minipigs, we used a porcine 4 × 44 K microarray chip containing 44,000 genes. Data normalization and clustering were performed using MA and scatter plots (Figure 2A,B) to reduce variation. The results showed 17,673 genes were upregulated and 22,575 were downregulated in the ovaries of conventional pigs (Table 1). In total, 40,248 differentially expressed genes were identified and further analyzed through hierarchical clustering. Among these, 1472 genes were more highly expressed in the conventional pigs, while only 681 were prominent in the minipigs. The significantly enriched pathways in the conventional pigs included those related to cell development, such as Wnt and IGF signaling. Hormonal signal pathways, including GnRH signaling, were also more active. Genes related to extracellular matrix (ECM) remodeling showed higher expression, suggesting more active tissue reorganization in conventional pigs. Gene ontology (GO) analysis showed increased expression across eight categories in conventional pigs, including two biological processes, two molecular functions, and four cellular components (fold-change cutoff = 2.0) (Table 2).

### 2.3. Significant Differences in Hormonal Action and Tissue Reorganization of Ovarian Tissue

**Figure 3:**
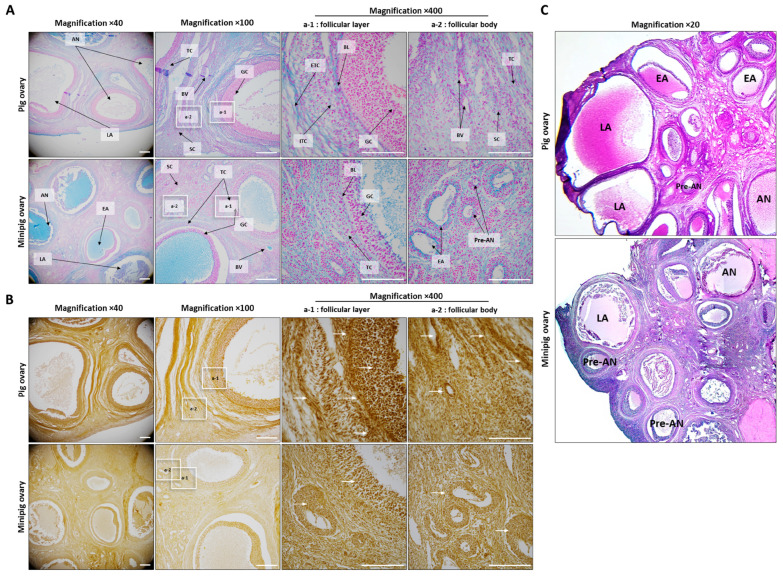
Analysis of physiological changes in the follicles of normal pigs and minipigs. (**A**): Alcian Blue staining was applied to ovary follicles to analyze the distribution of mucus in the follicles. (**B**): Alizarin Red staining was performed to analyze Ca^2+^ activity. (**C**): H&E staining. Abbreviations: AN, antral follicle; BL, basal layer; BV, blood vesicle; EA, early antral; ETC, extrathecal cell; GC, granulosa cell; ITC, intrathecal cell; LA, large antral; Pre-AN, preantral; SC, stroma cell; TC, theca cell. White arrows indicate representative high detection ranges. Scale bar = 100 µm; magnifications = 20×, 40×, 100×, and 400×.

**Figure 4:**
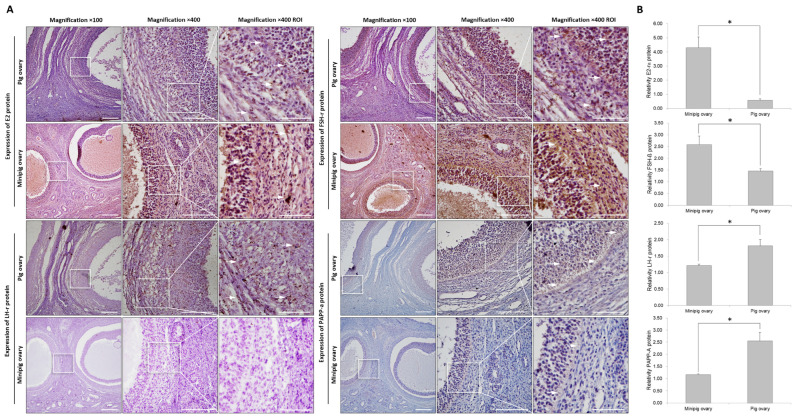
Comparative analysis of the expression locations and expression patterns of hormone-related factors. (**A**): Analysis of the expression patterns and localization of hormones responding to the follicles of normal pigs and minipigs. (**B**): Comparison of the expression patterns of hormones and PAPP-A in total ovarian protein using ELISA. White arrows indicate where the reaction is most pronounced. Abbreviations: ROI, region of interest. Scale bar = 100 µm; magnifications = 100× and 400×. * Different letters within the same column represent significant differences (*p* < 0.05).

Figure 1 and Figure 3C show clear morphological differences in follicle development between conventional pigs and minipigs. Figure 3A presents Alcian blue staining results for mucopolysaccharides. In the conventional pigs, the granulosa cell zone showed negative staining, while the theca cell zone showed a clear positive signal. In the minipigs, however, both granulosa and theca zones displayed positive staining, but the extra-thecal zone was much less developed compared to that of conventional pigs. In terms of calcium (Ca^2+^) activity, the ovaries of the conventional pigs showed strong activity throughout the tissue, especially in the granulosa and extra-thecal regions (Figure 3B). The minipigs displayed a similar pattern, but their overall Ca^2+^ activity was significantly lower. Figure 4 compares the expression and localization of E2, LH, FSH, and PAPP-A. All four hormones were primarily localized in the intra-thecal and granulosa zones, with little expression in the extra-thecal region. In the minipigs, E2 and FSH were more strongly expressed, particularly in the granulosa layer. In contrast, LH and PAPP-A levels were higher in the conventional pigs LH mainly in the intra-thecal cells, and PAPP-A levels were mainly higher in the granulosa zone. These differences were consistent and statistically significant.

### 2.4. Comparison of the Expression of Angiogenesis- and Tissue-Composition-Related Factors According to Differences in Follicle Formation

**Figure 5:**
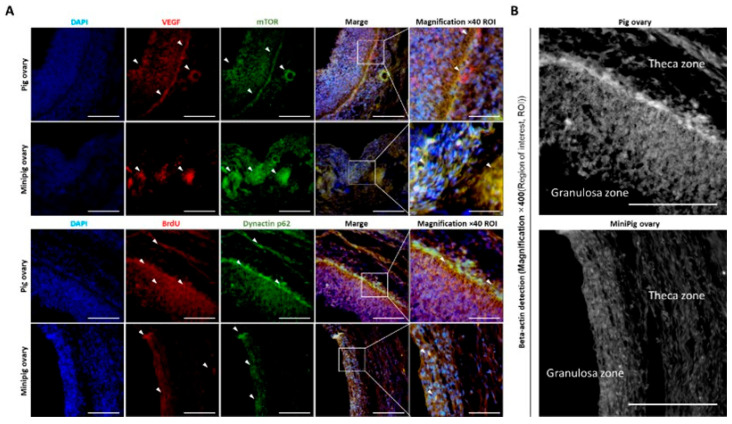
Comparison of the cell activity status of follicles by analyzing the expression patterns of cell-survival and angiogenesis factors. (**A**): The expression levels and localization of mTOR, VEGF, BrdU, and dynactin p62 proteins were analyzed using immunofluorescence, focusing on the granulosa and thecal zones of the follicles. (**B**): Analysis of cytoplasmic and morphological differences in tissues using β-actin fluorescent staining. White arrows indicate where the reaction is most pronounced. Magnification: 200x and 400x. Scale bar: 100 μm.

Figure 5 compares the expression of proteins involved in angiogenesis, cell viability, and tissue composition in conventional pigs and minipigs. In the conventional pigs, mTOR and VEGF were more strongly expressed in major follicular regions, consistent with the increased quantity of vascular structures observed earlier (Figure 1). BrdU and dynactin p62, both related to cell proliferation and cytoskeletal integrity, also showed higher expression in the conventional pigs. Likewise, β-actin staining indicated more developed granulosa and theca layers. We observed that gene expression related to vascular development and cell structure is generally reduced in minipigs, which may contribute to delayed follicular development and less efficient tissue remodeling.

## 3. Discussion

Minipigs have become invaluable in translational research on topics ranging from xenotransplantation to metabolic disease owing to their physiological similarities with humans [18,19]. However, we observed that follicular remodeling after corpus luteum formation in minipigs is marked by reduced VEGF-mediated angiogenesis and diminished IGF/AKT/mTOR survival signaling mechanisms [6], which were also shown to be disrupted by GnRH II receptor knockdown in gilts [20,21], collectively impairing follicle maturation and contributing to higher atresia rates, consistent with species-related differences in apoptosis during luteolysis [22].

In conventional pigs, robust VEGF expression fosters extensive capillary networks, enhancing progesterone delivery to enable optimal luteal support, whereas minipigs exhibit sparse vascularization and premature apoptosis via elevated Caspase-3 activity. These mechanistic differences parallel human luteal phase dysfunction documented in *Endocrine Reviews* [8], suggesting that minipigs may serve as models for studying impaired angiogenesis and survival signaling in luteal disorders. Thus, while minipigs mirror many aspects of human ovarian physiology, their unique regulatory network creates both opportunities to investigate luteal phase pathophysiology and challenges for developing breeding strategies that optimize fertility.

In our study, analysis of hormone responses and MMP activity after corpus luteum formation indicated that follicular regression in minipigs is associated with rapid apoptosis, particularly in the thecal layer, leading to reduced follicular function [20].

Our histological findings showed that the conventional pigs exhibited a well-developed extra-thecal region and a thicker granulosa layer, while the minipigs had less condensed granulosa cells and exhibited limited development in the extra-thecal zone. These results are consistent with prior reports linking corpus luteum formation to structural regression that supports luteal activity [10]. Additionally, we observed signs of follicular cell damage and apoptosis, such as disrupted micro-vesicles and elevated P53/Caspase-3 signaling alongside regulation of the IGF/AKT pathway, indicating that some follicular function is maintained throughout the estrous cycle in both species [21].

While both types of pigs showed activation of genes related to cellular structure and MMP-induced ECM remodeling [6,22], the minipigs displayed a notably slower rate of follicular development, which appears to be closely tied to increased apoptosis after corpus luteum formation [6]. Unlike previous studies that focused on follicular regression alone, our data suggest that some follicular structures remain hormonally active even after corpus luteum formation, possibly playing roles in secondary hormonal regulation [23,24]. Although minipigs have lower systemic estrogen levels and shorter estrous cycles, the minipigs in our study exhibited higher expression of estrogen and FSH receptors. In contrast, mTOR and VEGF signaling were significantly suppressed [24].

Our microarray results further supported this, showing lower expression of genes associated with cell viability, progesterone-mediated oocyte maturation, and ECM–receptor interactions in minipigs [9]. These results support the idea that minipigs undergo less active tissue remodeling and oocyte development. Hormonal signaling pathways in minipigs may be less responsive overall. Weak receptor activation and diminished feedback regulation likely contribute to reduced ovarian function. While earlier studies have suggested stable hormonal pathways [4], our findings show downregulation of cellular component genes and suppression of Wnt signaling, which may impair angiogenesis and follicular vascularization in minipigs [21,25].

Notably, we confirmed the presence of major hormone receptors in the granulosa cells regardless of corpus luteum status, and we observed localized VEGF expression around follicles, suggesting that vascular remodeling continues throughout the cycle [26]. However, expression of VEGF, mTOR, BrdU, and dynactin p62 was lower in the minipigs, indicating weaker regulation of cell proliferation and follicular remodeling. This suggests that while conventional pigs maintain some follicular activity post-ovulation, minipigs may experience functional exhaustion, contributing to lower fertility [23,26,27,28]. Taken together, our findings suggest that the estrous cycle and follicular development in minipigs are regulated by a more fragile hormonal network, leading to increased follicular atresia and impaired ovarian remodeling. The combined downregulation of VEGF and Wnt signaling, along with elevated apoptosis through Caspase-3 and P53, points to a distinct pattern of follicular regression in minipigs. This likely contributes to their reduced reproductive efficiency. Future studies should explore strategies such as hormonal supplementation or gene-based interventions to improve follicular survival and enhance reproductive outcomes in minipigs. Such research may also yield valuable insights for veterinary and biomedical applications.

## 4. Materials and Methods

All animal procedures were conducted in accordance with the National Guidelines for Agricultural Animal Care and were approved by the Hankyong National University Animal Experimental Ethics Committee, Korea. All immunological experiments were performed as described previously (HK-2023-2).

### 4.1. Collection and Preparation of Estrus Ovaries from Normal Pigs and Minipigs

Ethical approval (HK-2023-2) was obtained from the HKNU Animal Experimental Ethics Committee. Estrous cycles were monitored daily by evaluating standing reflex and vulvar morphology, with day 0 marked at first confirmed estrus. On day 15, corresponding to the mid-luteal regression phase cycle, staging was verified by measuring progesterone concentrations and inspecting ovarian structure, including counting corpora lutea and measuring average follicle diameters [27,28,29]. Under sterile conditions at Dodram LPC (Anseong, Republic of Korea), ovaries from five Yorkshire sows (10 months old; ≥3 cycles completed) and five Midget minipigs (10 months old; puberty onset at 3 months; ≥3 cycles completed) were harvested within 30 min of slaughter. Collected tissues were immediately submerged in precooled isopentane over liquid nitrogen and stored at –80 °C. For downstream analyses, luteal tissue was excised under a stereomicroscope, and remaining follicular ovarian tissue was divided for RNA extraction (TRIzol reagent), protein isolation (RIPA buffer), and paraffin embedding following fixation in 70% DEPC–ethanol with 5 µm sectioning.

### 4.2. Ovulation Rate Assessment

Corpora lutea were counted on hematoxylin–eosin-stained ovarian sections (5 µm) by two independent observers, following the method reported by Howard et al. [4]. The mean number of corpora lutea per ovary was calculated for each animal and used as an index of ovulation rate. These data are presented in Figure 1D.

### 4.3. Microarray

Total RNA was extracted from the ovary (without corpus luteum) tissues of normal pigs and minipigs using TRIzol reagent (Invitrogen, Waltham, MA, USA) according to the manufacturer’s instructions. Reverse transcription of the first complementary DNA strand was performed using oligo (dT) primers (Invitrogen). Porcine glyceraldehyde-3-phosphate dehydrogenase was used as the internal control. Microarray analysis was outsourced to a commercial provider using a porcine 4 × 44K chip (Chip no. 2526440, Agilent Technologies Inc., Santa Clara, CA, USA).The NCBI Gene Expression Omnibus (GEO) accession number for the microarray data reported in this study is GSE269724. Data are available at http://www.ncbi.nlm.nih.gov/geo/ (accessed on 1 Jun 2024), where readers can find a Supplementary Analysis File containing all preprocessing analyses, annotated lists of differentially expressed genes with links to NCBI, and gene ontology and pathway analyses (http://www.ncbi.nlm.nih.gov/geo/query/acc.cgi?acc=GSE269724, accessed on 15 June 2024). The images obtained were normalized and clustered using Agilent Gene Spring Software version 2 (Agilent Technologies Inc., Santa Clara, CA, USA). Cluster analysis of the molecular characteristics and biological information on cattle genes was performed using the KEGG PATHWAY database (http://www.genome.jp/kegg, accessed on 10 June 2024, Kyoto, Japan).

### 4.4. Histology of the Ovarian Tissue

Ovarian tissues of the normal pig and minipig groups were collected and fixed in 70% diethyl pyrocarbonate–ethanol, dehydrated, paraffin-embedded, and sectioned at 5 µm thickness. Representative sections from each ovary paraffin block in the treatment group were randomly selected, and routine hematoxylin-and-eosin staining and 4′,6-diamidino-2-phenylindole (DAPI) fluorescence (V11324, Thermo Fisher Scientific Solutions, Waltham, MA, USA) staining were performed to allow histological inspection under an optical microscope (×200, ×400) [6].

### 4.5. Alizarin Red and Alcian Blue Staining

Calcium and mucopolysaccharides in the ovarian tissue sections from each treatment group were analyzed using Alcian blue and Alizarin red S (ARS) (Sigma, St. Louis, MO, USA). First, the tissues were deparaffinized and hydrated to remove the antigens in the tissues and then immersed in 0.5% ARS (*w*/*v* in water; pH 6.36–6.4) to induce staining for approximately 30 min at room temperature. Subsequently, the sections were immersed in deionized water for approximately 5 min to stop staining, dehydrated in EtOH, fixed, and mounted with Permount. Calcium deposition was visualized by using an NI2-U (Nikon, Tokyo, Japan) to analyze orange and red spots. Histological analysis was performed using NIS-Elements C software (ver. 3.2).

### 4.6. In Situ Zymography

In order to perform an in situ Zymography experiment, deparaffinization/hydration was carried out twice in xylene, 100% ethanol, and 95% ethanol for 10 min, followed by washing in ddW for 5 min and boiling in 10 mM sodium citrate for 10 min. After that, an emulsion (ddW, 10% SDS, and 2% Glycerol) and a zymography reaction buffer were mixed in a 1: 2 ratio and placed on a slide; this was followed by enzymatic reaction at 37 °C for 48 h in a slide box filled with 1 M Tris. After the reaction, the slide was dried at 37 °C and stained with hematoxylin and eosin [29]. Then, the slide was covered with cover glass and analyzed with an optical microscope (×200, ×400).

### 4.7. Immuno-Detected Ovarian Tissue Protein

E2-rɑ (ab32063, Abcam, Cambridge, UK), FSH-β (sc-7797, Santa Cruz, Dallas, TX, USA), LH-r (ab76902, Abcam), and PAPP-A (sc-365226, Santa Cruz, Dallas, TX, USA) primary antibodies and proteins were placed on 96-well ELISA plates and activated at 4 °C for 1 day to evaluate specific proteins in each tissue. Next, immune reactions were induced using secondary antibodies (Rabbit and Mouse IgG-HRP antibodies, Abcam) for 2 h at room temperature, and a substrate solution (R&D Systems, Minneapolis, MN, USA) was added. The absorbance was measured at 450 nm.

### 4.8. Immunohistochemistry

Ovarian tissue slides were deparaffinized and hydrated. Deparaffinization and hydration were performed by removing each piece of paraffin from the xylene twice for 10 min, treating it twice in 100% and 95% EtOH for 10 min to remove the xylene, and then treating it twice in D.W for 5 min to complete the hydration process. The prepared ovary tissue slides were placed in 10 mM sodium citrate, heated for 10 min to retrieve antigens, and cooled at room temperature for 20 min. Endogenous peroxidases were inactivated with 3% H_2_O_2_ for 5 min and washed thrice with 1×PBS for 5 min each. The processed slides were blocked with 5% NHS + 1% normal goat serum 1×PBS for 1 h. Then, the blocking solution was removed, and the primary antibodies were diluted 1:200 in the blocking solution and incubated overnight at 4 °C to induce antigen-antibody reactions. The slides were washed with 1×PBS for 5 min, and the secondary antibodies, HRP-conjugated anti-rabbit and anti-mouse (IgG-HRP antibody, Abcam), were diluted 1:200 in the blocking solution and incubated for 1 h at RT. The slides were then washed thrice for 5 min each with 1×PBS. Afterwards, the slides were reacted with ABC reagent (Vector laboratories, Newark, CA, USA) for 30 min, washed thrice with 1 × PBS for 5 min each, reacted with 300 µL of DAB (Vector laboratories, Newark, CA, USA) for 1 to 10 min, treated with deionized distilled water for 5 min to stop color development, counterstained with hematoxylin, and then dehydrated by being treated twice with 95% and 100% EtOH and xylene for 10 min each. The stained slides were mounted using Permount and observed under an NI2-U microscope (Nikon, Tokyo, Japan).

### 4.9. Immunofluorescence

The expression of specific proteins in ovary tissues was analyzed via immunostaining in accordance with previously reported protocols [9]. Ovary tissues collected on day 15 were deparaffinized following the immunohistochemistry protocol, rinsed in PBS for 30 min, and permeabilized with 0.2% Triton X-100 for 30 min at room temperature. After being blocked with 3% bovine serum albumin in PBS, the samples were incubated with antibodies against the active forms of mTOR (#2972, Cell Signaling Technology, Danvers, MA, USA), VEGF (ab150375, Abcam), BrdU (ab152095, Abcam), and dynactin p62 (sc-25730, Santa Cruz Biotechnology, Dallas, TX, USA) in 1:300 dilution. Subsequently, the samples were washed and incubated at room temperature for 2 h with secondary antibodies, namely, Alexa Fluor™ 488 (A11001, Invitrogen, Waltham, MA, USA) and goat anti-rabbit IgG (H+L) Dylight 594 (35560, Invitrogen), both in 1:300 dilution. Nuclei were counterstained with DAPI (Sigma, St. Louis, MO), and the samples were mounted using a fluorescence mounting medium. Finally, the cells were imaged using a fluorescence microscope (NI2-U; Nikon, Kyoto, JPN).

### 4.10. Statistical Analysis

ELISA and micro-array data were tested for significance (Duncan and General Linear Model) using Statistical Analysis System software (SAS Institute, version 9.4, Cary, NC, USA). Statistical significance was set at *p* < 0.05.

## 5. Conclusions

In this study, we investigated the molecular and hormonal differences in follicular development between minipigs and conventional pigs. The results show that minipigs have weaker hormonal signaling, reduced expression of vascular and growth-related genes, and a higher rate of follicular cell apoptosis. These factors likely contribute to their lower reproductive efficiency. Compared to conventional pigs, the minipigs showed less active ovarian remodeling after corpus luteum formation. While mTOR and VEGF were highly expressed in the conventional pigs, these pathways were downregulated in the minipigs. Histological findings supported this, revealing poorer follicular structure and reduced vascularization. The data suggest that minipigs operate under a more fragile endocrine network, which may not support follicular maintenance as effectively. Although this study focused on gene expression and tissue morphology, further work is needed to evaluate hormone receptor function and other regulatory pathways. Finally, this work points to possible areas for intervention in regard to hormonal treatment, genetic targeting, or adjusted breeding conditions. These approaches could help improve follicular survival and fertility in minipigs, an especially important goal given their growing role in biomedical research.

## Figures and Tables

**Table 1:** Analysis of DOWN and upstream patterns of genes subjected to RNA microarray analysis.

Analysis Design	Cutoff	Regulation	Significant Gene (No.)	GO Information (No.)	Pathway Information (No.)
Pig_ovary vs. Minipig_ovary	1.3	DOWN	11,427	1590	1356
		UP	8247	2570	2218
	1.5	DOWN	7290	1024	882
		UP	5692	1958	1743
	2.0	DOWN	2923	486	399
		UP	2694	1017	945
	3.0	DOWN	935	195	147
		UP	1040	455	399

The darker the orange, the higher the number of genes with significant differences.

**Table 2:** Analysis of DOWN and upstream patterns of genes subjected to RNA microarray analysis.

GO Category ^(1)^	Category ^(2)^	Total Genes ^(3)^	Changed Genes ^(4)^	Enrichment ^(5)^	Log10(p) ^(6)^	*p*-Value ^(7)^	False Discovery Rate ^(8)^
GO:0055114_oxidation_reduction	biological_process	130	68	2.019967	−10.688729	2.05 × 10^−11^	0
GO:0016491_oxidoreductase_activity	molecular_function	158	72	1.759763	−7.786661	1.63 × 10^−8^	0
GO:0005739_mitochondrion	cellular_component	96	47	1.890625	−6.292212	5.10 × 10^−7^	0
GO:0009055_electron_carrier_activity	molecular_function	60	33	2.123936	−5.989606	1.02 × 10^−6^	0
GO:0005737_cytoplasm	cellular_component	502	170	1.307748	−5.796112	1.60 × 10^−6^	0
GO:0044444_cytoplasmic_part	cellular_component	343	123	1.384809	−5.464044	3.44 × 10^−6^	0
GO:0044429_mitochondrial_part	cellular_component	55	30	2.106383	−5.385052	4.12 × 10^−6^	0
GO:0022900_electron_transport_chain	biological_process	22	16	2.808511	−5.304345	4.96 × 10^−6^	0

(1) GO category: GO number and GO term. (2) Category: top-level category to which GO belongs; BP: biological process; CC: cellular component; MF: molecular function. (3) Total genes: the number of genes in the input microarray data belonging to the corresponding GO. (4) Changed genes: the number of genes whose expression values in the experimental group are significantly different from those in the control group among all the genes in the corresponding GO (fold-change cutoff = 2.0). (5) Enrichment: the degree to which significant genes in the corresponding GO are enriched. (6) Log10(p): the value obtained by taking the log2 of the *p*-value of Fisher’s exact test results indicates whether the number of significant genes in the corresponding GO is statistically significant. (7) *p*-value: the *p*-value of Fisher’s exact test indicates whether the number of significant genes in the corresponding GO is statistically significant. (8) False discovery rate: a value indicating the possibility that the corresponding GO is false positive.

## Data Availability

The additional data supporting this manuscript are available from the corresponding author upon request.
